# MolQuery: Prediction of Lipid Synthesizability Using Active Learning

**DOI:** 10.1021/acsomega.5c09931

**Published:** 2026-02-11

**Authors:** Jonathan Broadbent, Jiří Vymětal, Saeed Moayedpour, Michael Bailey, Saleh Riahi, Akshay Balsubramani, Peter Mikochik, Luc Even, Naresh Gunaganti, Ramesh Dasari, Saswata Karmakar, Hongfeng Deng, Vikram Agarwal, Ziv Bar-Joseph, Sven Jager

**Affiliations:** † Digital R&D, 201256 Sanofi, Toronto, ON M5V1V6, Canada; ‡ DataSentics, 602 00 Brno, Czech Republic; § Digital R&D, 201256 Sanofi, Cambridge, Massachusetts 02141, United States; ∥ mRNA Center of Excellence, Sanofi, Waltham, Massachusetts 02451, United States

## Abstract

In the field of molecular design, Generative Artificial Intelligence (GenAI) has the potential to create extensive synthetic data sets encompassing a wide range of chemical properties. However, the practical application of these data sets is often constrained by the synthesizability of the molecules within. To address this, it is essential to develop a robust platform for assessing synthesizability, which is crucial for constructing effective GenAI-based models for molecular systems. Here, we introduces MolQuery, a comprehensive pipeline that integrates active learning (AL) to improve the accuracy of chemical synthesizability predictions for lipid molecules designed for mRNA delivery via lipid nanoparticles (LNPs). By leveraging AL, MolQuery efficiently trains machine learning models using small data sets which greatly improves upon current solutions for this tasks. Our results demonstrate that MolQuery produces highly accurate predictions of lipid synthesizability, making it a valuable tool for filtering synthetic LNP data sets.

## Graphical abstract

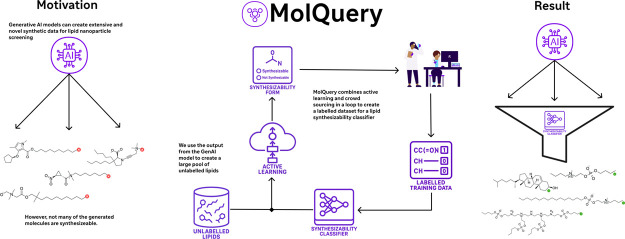

Lipid nanoparticles (LNPs) are an essential component of mRNA vaccines, encapsulating the charged mRNA molecule and facilitating their delivery to cells via membrane fusion. All currently approved therapeutic LNPs include four lipids: an ionizable cationic lipid and three helper lipids 1,2-Distearoyl-*sn*-glycero-3-phosphocholine (DSPC), cholesterol, and a polyethylene glycol (PEG)-lipid conjugate.^1–3^ The selection of lipid components determines the vaccine’s efficacy and stability in the bloodstream, while also modulating inadvertent immunological reactions and triggering apoptotic and inflammatory responses.^4^ Additionally, the structural features of lipids can be used to enable the targeted delivery to specific cells, such as T lymphocytes.^5^

Ionizable cationic lipids are the most critical components of LNPs, and significant effort has been dedicated to optimizing their properties over the past 20 years. Given their large structure and complex physiochemical properties, computer based-algorithms for de novo lipid design have faced significant challenges. The large combinatorial space including the options for selecting the ionizable group, pH dependency, the tail region and the identity of branches make engineering a lipid with optimal properties very difficult.^6–8^

A first step toward computer based molecular design (including for LNPs) is to ensure that the proposed molecule is easy to synthesize.^9^ Given the large amounts of lipids that are needed for commercial vaccines, the cost and difficulty associated with chemical synthesis/purification is a critical consideration for selecting a molecule.^10^ Moreover, reducing the number of synthetic steps could lower the prevalence of impurities and side products which improve the homogeneity of formulated LNPs.^11^ Given the importance of this problem, several recent models and scoring schemes have been devised for synthesizability prediction.

DeepSA^9^ is a deep learning model based on a natural language processing (NLP) technique that takes the SMILES representation of a molecule and attempts to characterize how easily a compound can be synthesized using available methods and resources. DeepSA can leverage a variety of chemical language models as the encoding layer, including ChemBERTa-77M-MTR (ChemMTR).^12^ Another method, SAScore^13^ uses the Extended Connectivity Fingerprint (ECFP) to evaluate and score molecules with respect to their synthesizability. The method starts with a very large number of randomly selected compounds from the PubChem database.^14^ Following ECFP fragmentation, a scoring scheme is used to score the fragments based on their frequency in the data set (more prevalent fragments are considered more accessible for synthesis). A heuristic penalty on molecular complexity is applied to penalize molecules with stereocenters, macrocycles, spiro, and bridge atoms. Similarly, SYBA^15^ (Synthetic Bayesian Accessibility) is another fragment-based method utilizing Extended-Connectivity Fingerprints (ECFPs). It employs a Bernoulli Naïve Bayes classifier to assess the synthesizability of each fragment within a molecule. The classifier assigns a score to each fragment based on its frequency in a database. GASA (Graph Attention-based assessment of Synthetic Accessibility)^16^ uses a graph neural network (GNN) with an attention mechanism to autonomously identify the key structural features relevant to synthetic accessibility. By sampling near the hypothetical classification boundary, GASA enhances its capability to differentiate between structurally similarity. Finally, the Retrosynthetic Accessibility Score (RAscore)^17^ is a ML-based metric designed to quickly assess the synthetic feasibility of chemical compounds. It leverages predictions from computer-aided synthesis planning (CASP) tools, such as AiZynthFinder,^18^ to determine whether a synthetic route can be identified for a given molecule.

While many machine learning methods for predicting transfection efficiency or stability have been developed so far, a model for synthesizability of LNPs has not yet been established.^19–21^ Moreover, methods for retrosynthetic approaches do not cover the synthesis of lipids and are applied more to small molecules in general.^22^ Thus, synthesizability models fail for lipids due to their unique structural characteristics and the complexity of their synthesis. Traditional models do not account for these differences, and the synthesis of lipids involves more steps and different types of chemical reactions. Additionally, the limited data available for lipid synthesizability makes it challenging to train accurate models. Gathering lipid synthesizability data can fill this gap and enable the synthesis of more complex lipid structures.

While simply labeling a large data set of potential LNPs may lead to a good training data, a more efficient way to achieve this is to use Active Learning (AL). AL is a framework for generating data for cases where labeled data is scarce.^23^ Following initial training, AL selects the most informative batch of data from the pool for labeling. Models trained with an AL protocol exhibit better performance and are more generalizable for a fixed amount of labeled data.^24^

To improve the accuracy of lipid synthesizability prediction and to provide new labeled data for training we developed a two step approach. Our method uses a large language model (LLM; i.e. Claude-3-Sonnet)^25^ to generate a diverse set of ionizable lipid molecules. AL is then used to select a subset of these for labeling. We developed an interactive UI (Figure 2) allowing expert chemists to assign binary labels to selected molecules. The number of synthesis steps that define synthesizability we leave as a hyperparameter of the laboratory to determine. The data is then used to train a classifier and the process iterates until convergence. As we show, using both the generative model and the AL approach led to significant improvements over current methods for lipid synthesizability prediction allowing scientists to save valuable time when testing new lipids (Figure 1).

**Figure 1:**
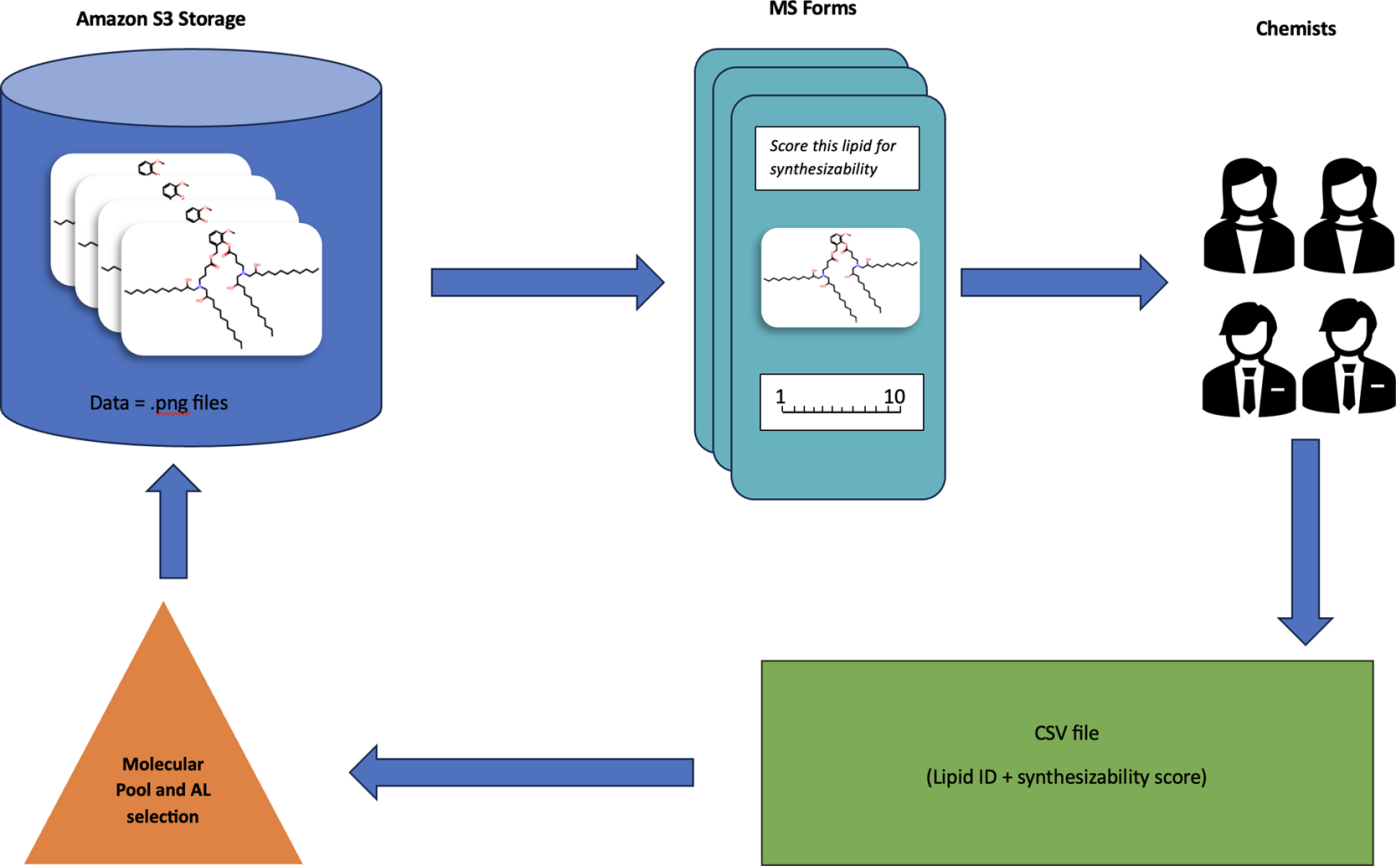
MolQuery Pipeline. A large pool of unlabeled lipids is generated using an LLM, we use AL selection to select molecules for labeling. Images are stored in an S3 bucket, forms are then populated by Power Automate and shared with chemists on Sharepoint. Results are saved to a CSV file and used to retrain the synthesizability model in the next iteration.

## Results

We developed a lipid synthesizability prediction pipeline that employs AL as a strategy for optimal annotation of new data to train the underlying ML model. In this study we utilized the CatBoost classifier, which we selected based on its performance in predicting the transfection efficiency of lipids.^26^ The pipeline includes an associated labeling campaign, which collects ground truth labels assigned by field experts. Due to the substantial cost of time needed by experts to manually label the lipids, the number of samples in the training data set is restricted, making their optimal selection highly important.

### Collecting Synthesizability Data

In total, five batches of AL were conducted based on selections from computationally generated molecular pools. The basic characteristics of the entire data set, including the initial internal data set and the subset selected through AL, are shown in Table 1. The total data set is roughly balanced in terms of the counts of positive and negative classes; however, the subset from AL was found to be much more biased toward positive classes. A more detailed view of data collection during the AL phase is provided in Table 3.

**Table 1:** Basic Data Set Characteristics

stage	samples	positive	negative
total data set	257	155	102
initial part	160	76	84
active learning part	97	79	18

The questionnaire form designed for annotation of lipid molecules is shown in Figure 2. The web interface includes a graphical panel displaying the assessed molecule, input text boxes for annotations and comments, and a panel with detailed instructions. Annotators could freely browse the list of assessed molecules and submit their evaluations in any order they preferred. Completing all forms was not compulsory; annotators were free to skip any molecules if they were unsure about their evaluation.

**Figure 2:**
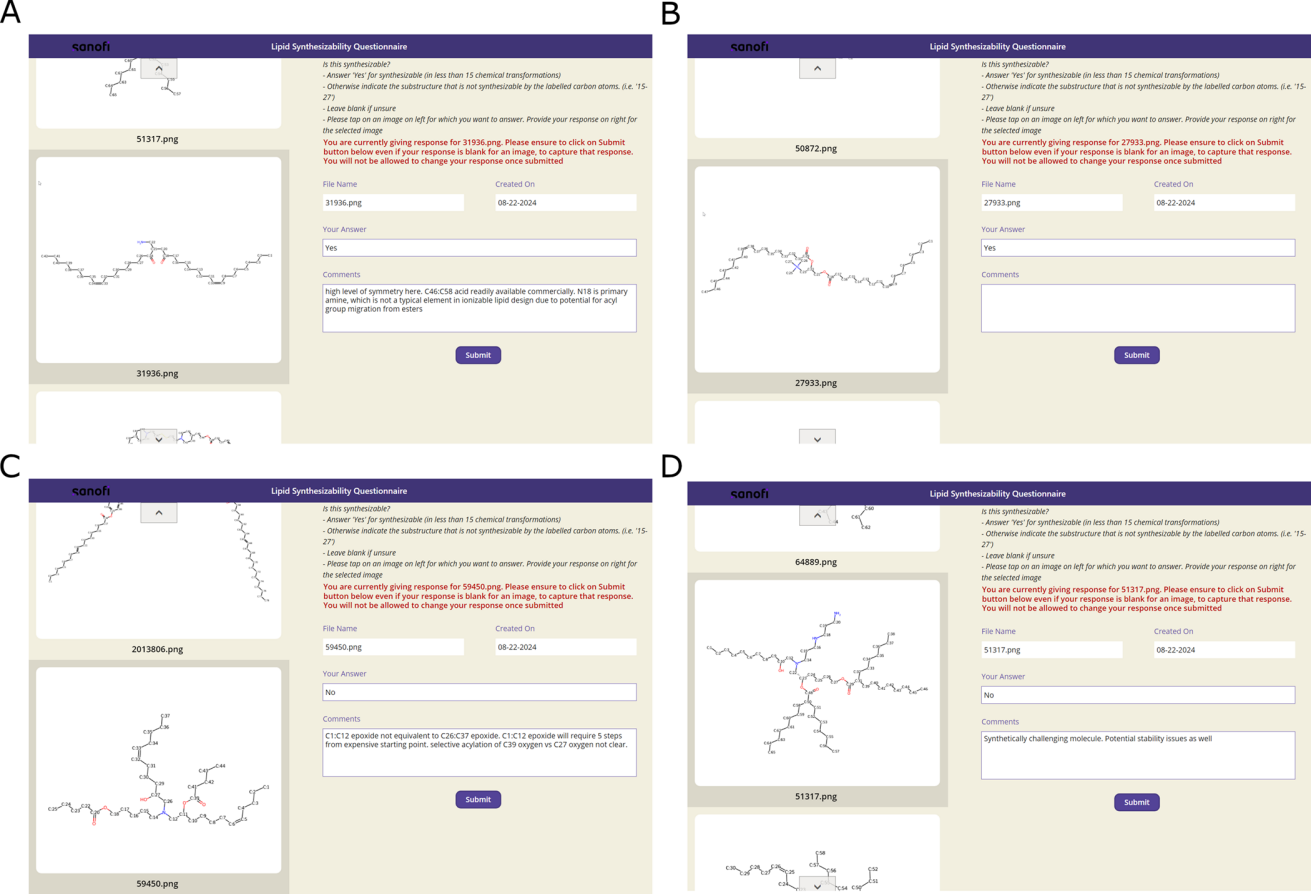
Questionnaire for synthesizability assessment. The figure displays four panels with screenshots of the questionnaire used for synthesizability assessment. The main components of the questionnaire form include the structural chemical formula of the assessed molecule, an input box for the final decision, and an input box for comments and explanations supporting the decision. Panels A and B show molecules that were classified as synthesizable, while Panels C and D present examples of molecules that were marked as unsynthesizable. Molecules in panels A and B have been replaced with placeholders for intellectual property reasons.

We used a batch size of 30 molecules in each active learning round, the number of labeled molecules in each round was approximately 20 but varies due to the availability of annotators. As shown in Table 3, only 20% of the lipids were nonsynthesizable, resulting in a skewed data set. Nevertheless, because the selected lipids maximize the AL acquisition criteria, each label contributes to improving the model. We generated three different unlabeled lipid pools using a combinatorial method and generative AI (further described in the methods section). A major challenge in generating the pool for the AL selection was variability. The variety of the original combinatorial library (”comb”) dropped significantly after filtering out compounds with synthesis-challenging chemical moieties, as identified by expert chemists during the labeling campaign. Therefore, we moved to using LLM-generated compounds (”llm1”). The final change to the pool aimed to decrease the molecular weight of the unlabeled molecules, as the LLM model had generated molecules with weights skewed toward the upper limit of the acceptable range. Consequently, an additional set of lipid molecules was generated by the LLM model with modified criteria, enriching the pool (”llm2”) for the last two rounds of AL batches. See the Supporting Information for a detailed description about the construction of “llm1” and “llm2” pool.

### Annotation Consistency

Table 2 provides information on annotation redundancy. To maximize the throughput of our labeling campaign, the majority of the molecules received only a single annotation. However, to measure consistency of annotations we assigned 23 molecules to two independent annotators, and seven molecules to three annotators. Among the 30 molecules, there was one disagreement. Therefore, we infer 96.7% agreement in annotations. In the case of the single spurious annotation, we chose the final annotation based on the provided reasoning.

**Table 2:** Annotation Statistics

count of independent annotations	count of samples
1	227
2	23
3	7

### Active Learning Performance

The performance of the ML model was monitored using standard metrics for binary classifiers: accuracy, AUPRC, AUROC, and F1 score. The evolution of model performance during AL, measured using a consistent train/test split strategy, is shown in Figure 3. As can be seen, all metrics indicated increasing prediction quality following the application of AL, up to the third batch improving the accuracy from 0.64 to 0.72, F1 score from 0.67 to 0.78, AUPRC from 0.80 to 0.83 and the AUROC from 0.72 to 0.76. For the last two batches, all metrics plateaued, indicating no further improvements indicating that 5 rounds are enough to obtain an optimal model.

**Figure 3:**
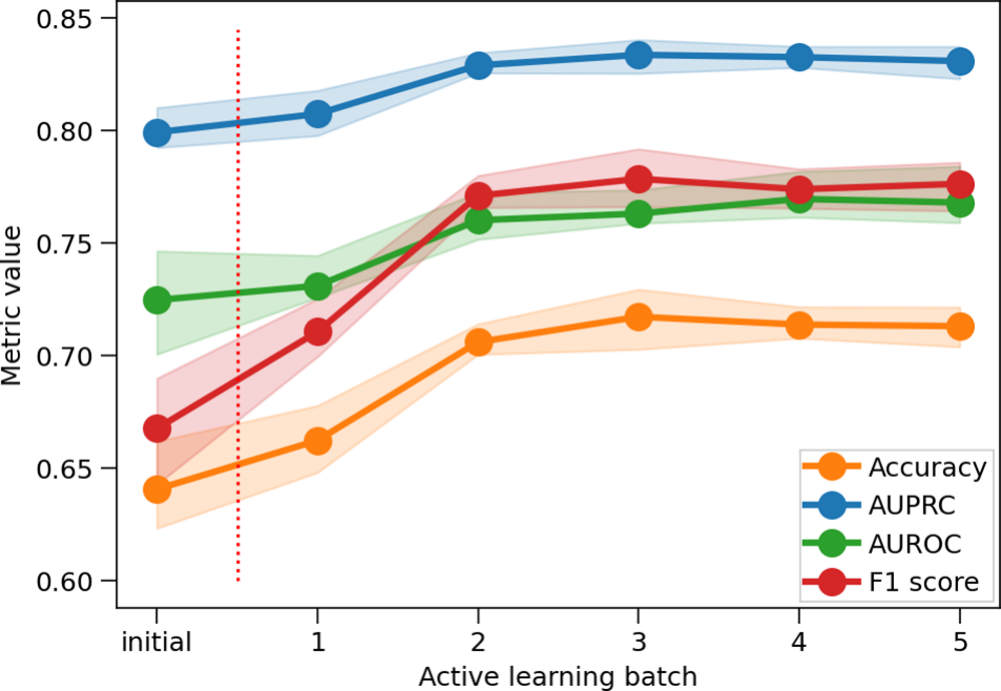
Performance of the CatBoost model on the data set using AL. The evolution of accuracy, AUPRC, AUROC, and F1 score is shown, along with intervals delimiting the 20th to 80th percentiles of independent cross-validation estimates for these metrics. The statistics are collected on ten independent train/test splits.

### Retrospective Experiments

We conducted retrospective experiments to compare active learning (AL) with random sampling using a labeled data set of 257 lipids (Table 3). Across 200 experiments, a CatBoost classifier was iteratively trained on AL-selected batches and evaluated on a fixed test set to assess performance gains over random selection (Figure 4). The distribution of data set sizes needed to achieve the targeted accuracy shows a significant bias toward smaller sizes for the AL strategy, indicating faster convergence when using AL. The difference between the distributions is significant, with a p-value of 0.004, as evaluated by a chi-squared contingency test. On average, the data set size needed to reach the targeted accuracy was 68 samples for the AL and 85 samples for random sampling. The corresponding median data set sizes are 65 and 75, respectively. This demonstrates that random selection requires more data to achieve the same accuracy as AL.

**Table 3:** Progress of the Annotation Campaign during AL Phase

batch	labeled	unlabeled	positive	negative	pool
1	20	10	18	2	comb
2	16	14	15	1	llm1
3	19	11	12	7	llm1
4	20	10	13	7	llm2
5	22	8	21	1	llm2
total	97	53	79	18	

**Figure 4:**
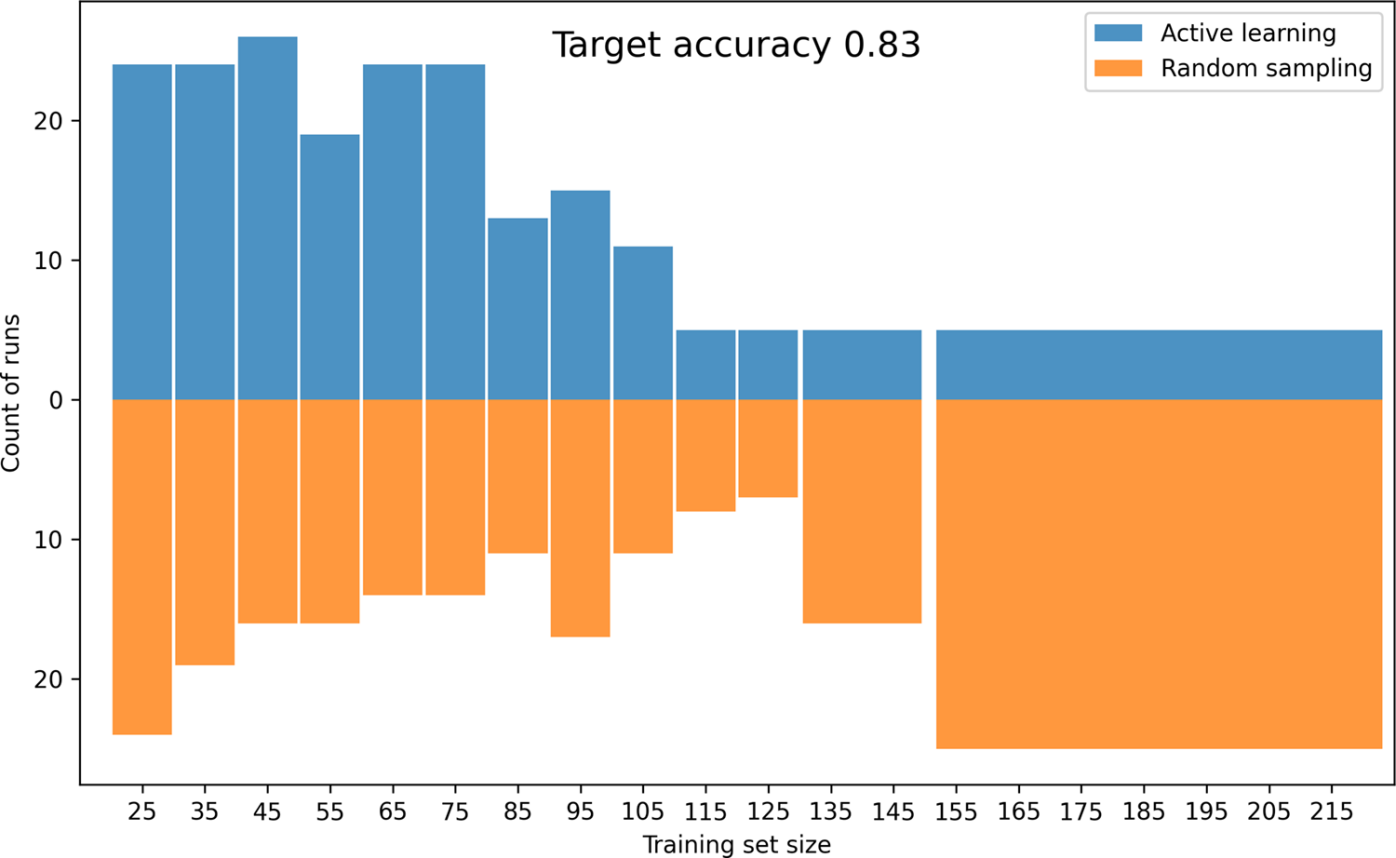
Retrospective experiment comparing active learning (AL) with random selection. The plot shows the distribution of data set sizes (from 200 replicas) at which the predictor first achieved an accuracy of 0.83 on the test set the benchmark corresponding to a model trained on the full training data set. Notably, some replicas reached this threshold using only the initial set (first bin), independent of the selection strategy. The final two bins are widened to ensure inclusion of at least five samples from each distribution, satisfying the assumptions of common statistical tests.

### Lipid Synthesizability Benchmarking

Table 4 provides a comparison of our model with previously published methods for synthesizability prediction. The first two rows are different implementations of the classifier in our method while the other rows present results from prior methods. Each value is the mean and standard deviation of a 5-fold cross-validation. As can be seen, our method greatly improves upon other methods for lipid synthesizability predictions. We see improvements of between 20 and 40% for accuracy and 5–100% for F1 scores. The next best method is the heuristic-based SAScore followed by the deep learning method DeepSA.

**Table 4:** Lipid Synthesizability Benchmarking on Complete Dataset (5-Fold Cross-Validation)

model/method	accuracy	AUPRC	AUROC	F1
CatBoost (circular fingerprints)	**0.72** ± 0.03	0.84 ± 0.03	0.71 ± 0.03	**0.79** ± 0.04
CatBoost (LipoBart embedding)	0.71 ± 0.04	**0.87** ± 0.03	**0.78** ± 0.03	0.77 ± 0.02
SAScore	0.62 ± 0.05	0.79 ± 0.06	0.68 ± 0.06	0.68 ± 0.04
SYBA	0.40 ± 0.08	0.78 ± 0.05	0.62 ± 0.06	0.20 ± 0.06
DeepSA (SmELECTRA)	0.61 ± 0.06	0.79 ± 0.06	0.70 ± 0.08	0.75 ± 0.05
GASA	0.61 ± 0.06	0.78 ± 0.07	0.68 ± 0.07	0.75 ± 0.05
RAScore	0.61 ± 0.06	0.74 ± 0.05	0.61 ± 0.08	0.75 ± 0.05

Since the above models were only trained on small molecules, we also tested whether the new data obtained by our AL framework can help improve their performance if they were trained on that data as well. Table S1 in the Supporting Information indicates that incorporating lipid molecules indeed improved the predictions on the test portion of our data set within the used cross-validation scheme. However, for almost all of them the results were still lower than the results obtained by our CatBoost classifier. Only the variant with the DeBERTa encoding layer obtained results that are on par with the CatBoost results when using the AL data described in this paper. We note that retraining with lipid compounds did not negatively impact the performance of DeepSA variants on the original small molecule test data set. In fact, some metrics were improved (see Table S2 in the Supporting Information) indicating that the data generated by the AL approach described in this paper can be of general use for other chemical prediction tasks as well.

### Hyperparameter Robustness

We conducted a hyperparameter grid search on selected parameters to evaluate their impact on classification performance across the entire data set. Table S3 illustrates how the radius of the ECFP, its size in bits, and the inclusion of chirality and counts affects all metrics. Although their effects are relatively minor, the following trends were observed: including counts and chirality improves performance. Small radii (below three) result in slightly worse performance, while no further improvement is observed beyond a radius of three or four. The size of the fingerprint does not significantly affect performance. Similarly, Table S4 demonstrates that variations in the three most important parameters of the CatBoost classifier (number of iterations, learning rate, and tree depth) do not significantly influence performance.

## Discussion

We developed a pipeline for the AL with lipid molecules. The pipeline utilizes a pool of lipids generated by a state-of-the-art GenAI approach, combined with AL to select the most informative batch of compounds for labeling by chemical experts.

The need for improved prediction methods for LNPs is evident from the results presented in Table 4, which illustrates the failure of state-of-the-art methods for synthesizability prediction on this type of molecule. The chemical-fragment-based methods employing statistical approaches and heuristics (SAScore, SYBA) performed surprisingly well compared to deep learning predictors such as graph neural networks (GASA) and neural networks utilizing language model embeddings (DeepSA). The method based on predicting viable chemical synthesis routes (RAscore) showed the worst performance on our lipid data set.

Comparatively we see a large gain in performance using our AL curated data set. A limitation of this study is that the model is restricted to the space of generated lipids used during training, limiting generalizability to unseen lipid structures. Furthermore, the binary labels are specific to our laboratory. Here we defined feasible synthesizability as *k* = 15 steps. This is unlikely the case for all environments, however, we present this method as a framework to tailor make a synthesizability model for any given use case.

We used CatBoost as the predictive model for synthesizability. CatBoost offers efficient training speed combined with the robustness and interpretability of tree models. It also works well with ECFP fingerprints and delivers good performance with default parameters, without requiring extensive hyperparameter tuning. However, other options are possible. The Alien package for active learning supports interfacing with a variety of models, primarily neural network-based, implemented in PyTorch, Keras, or DeepChem.^23^

It is important to note that our CatBoost classifier is specifically fitted to lipid-like molecules, whereas the other methods target a broader range of chemical compounds, typically drug-like molecules and those found in chemical databases such as PubChem. The area of lipid-like molecules in the chemical space might not be sufficiently covered by current synthesizability predictors, as indicated by the results in Table 4. These evaluations indicate that the chemical space occupied by lipids lies outside the scope of current ML methods trained on small molecules, while some principles of their chemical synthesis are captured by simpler methods. We further validated this hypothesis by retraining DeepSA, proving its ability to improve the coverage the lipid chemical niche when lipid representatives are included in the training set.

AL improved the performance of the algorithm tested using the newly labeled lipids. While the set used was biased toward positive examples, performance still improved. This is evidenced by the rapid improvement in performance metrics when AL was employed (Figure 3). During the AL phase, these improvements eventually plateaued, indicating that the limits were reached and no further rise in performance metrics was observed. A more balanced set of positive and negative lipids for labeling may have led to an improved performance. Making sure the generative models can provide such sets is a direction for future work.

However, even with the new set of labeled lipids, none of the methods was able to achieve an accuracy above 75%, lower than those reported for other classes of molecule.^9,13,15–17^ To test potential causes for this observation we examined the log loss function used by CatBoost. The distribution of the log loss values is shown in Figure S1. We inspected the samples with the highest log loss values (Figure S2). Nine out of the top ten outliers belong to a single scaffold with similar functional groups attached at various positions. These nine lipids were classified differently by individual chemists (i.e., some were determined to be synthesizable while others were determined not to be). Thus, a likely cause for reduced performance is issues related to the subjectivity of the labeled data. Future work should focus on further improving the labeled data.

The analysis we performed reached the limit given the data we used. More rounds could only help if the accuracy of the labeled data increases or if a more diverse lipid pool can be sampled and chosen. Conditioning a generative model on synthesizability labels to ensure well-distributed data is essential. Future work should use such an approach to explore a wide range of lipid structures and improve the robustness of the model. Other issues that can be addressed in future work include:

- Inconsistencies in labeling. As noted above, we cannot exclude the possibility that some molecules are mislabeled. The focus of this study was to generate a good synthesizability data set so we directed our resources toward maximizing the number of labels created. We performed a consistency analysis among 30 redundant annotations and saw 96.7% agreement between chemists’ annotations. Future work could select molecules with high log loss for relabeling to improve reliability.
- Continuous Scoring. In this work, we created a labeling scheme that defines synthesizability with binary labels defined by a *k* = 15 steps cutoff threshold. This was a solution tailored to our laboratory. We used a binary labeling scheme to simplify the task of the ML classifier. Future work could experiment with using a continuous scoring scheme and a classifier which predicts the number of steps for any given molecule. This would enable the tool to generalize to environments beyond the given laboratory.
- Representation of lipid molecules for ML. Similar to previous work focused on predicting the transfection efficiency of lipids forming LNPs,^26^ we observed mild performance gain using the LipoBart embedding, however, it did not beat completely the baseline ECFPs in all metrics. Additionally, most of the other language models incorporated into DeepSA did not further enhance performance upon retraining. Several scenarios are plausible: either the ECFPs are performing extraordinarily well for this task, capturing the necessary signals for prediction, or alternatively, all these methods fail to identify advanced features for synthetic accessibility predictions, thus hindering further improvements.

Lipids, by their nature, consist of various chemical moieties connected by aliphatic fragments of different, often larger, sizes. Their respective positions in the molecule can provide valuable information; however, it is questionable whether these features are extracted by current molecular representations. See Supporting Results for more discussion.

## Conclusions

In this work, we designed a specialized pipeline for training a synthesis accessibility classifier for bioactive lipid molecules. This pipeline utilizes a state-of-the-art GenAI approach for the automated high-throughput design of novel molecules with desired properties and AL for optimal batch selection, labeled by experts in medicinal chemistry. In this pilot study, the pipeline generated an annotated, diverse data set of lipids, on which current synthetic accessibility classifiers showed suboptimal performance, being outperformed by a dedicated CatBoost classifier. We thus demonstrated the limited transferability of synthetic accessibility classifiers to novel classes of molecules outside their parametrization set. The presented pipeline is intended to overcome these limitations and serve as a valuable complement to other tools in screening procedures for drug development and lead optimization.

## Methods

### Labeled Synthesizability Data

We first set out to obtain a labeled data set for lipid synthesizability. For this we used both, prior data and obtained new labeled data based on expert evaluation. In the initial phase, our internal data sets of lipids were assessed for their synthesizability. The second phase involved AL to select the next set of lipids to label. For this phase, a targeted pool of computationally generated unlabeled molecules was employed.

Labeling was conducted in several batches, a group of expert medicinal chemists, were asked to assess 30 molecules. The experts were asked to assess the synthesizability of the chemical compounds using an online questionnaire with detailed instructions for labeling. Chemists were asked to estimate the number of reaction steps required to synthesize each molecule. If the estimated number of reaction steps was fewer than *k*, the molecule is labeled as synthesizable; otherwise, it is labeled as nonsynthesizable.

We leave *k* as a hyperparameter for this method to be set according to the use case. Defining a synthesizability limit should take into consideration limits on waste products, resource time, value of product and overall yield. In our case, we set the tool up for our laboratory that defined *k* = 15 as feasible for synthesis. This approach enables one to tailor the method toward a given application. We recommend to set *k* ≤ 25 to ensure workable overall yields.^27–29^

In addition to the questionnaire, annotators were encouraged to contribute to another form that maintained a list of specific chemical moieties hindering easy synthesis. This list of synthetically challenging moieties was regularly used to prefilter the molecular pools of unlabeled lipids, thereby avoiding repetitive annotation of compounds containing them.

### Architecture of the Questionnaire System

Images of molecular structures were drawn with the RDKit Draw module^30^ and stored in an S3 storage bucket (Figure 1). We used Power Automate to automatically create and populate Microsoft forms on Sharepoint once a week. The forms were sent out to a list of subject matter experts for review. Upon completion of any part of the form, developers were notified and the responses were collated into an excel file. At the end of the week the responses were aggregated with those from previous labeling pools into a CSV file with all of the responses. We would then use the new data set to train our synthesizability model. With our new metrics we selected a new batch of molecules for labeling based on those that are most likely to improve the model (see section on Active Learning).

### Molecular Pool

#### Initial Combinatorial Pool

To generate a large pool of lipid molecules we first curated a database of structures published in scientific literature.^31^ We then applied a few known linker-associated reactions^32^ to decompose them into lipid structure fragments in-silico. With the large fragment library, we recombined all the fragments using the same linker-associated reactions for in silico synthesis of a rich sampling of different combinations of the fragments, with chemically plausible linkers. This process yielded over 100,000 lipids, almost all (>99%) of which are unexplored and not previously synthesized.

#### Using LLM to Generate Lipid Libraries

The additional pool of molecules was generated using a “Many-Shot In-Context Learning” approach^33^ with the LLM model Claude Sonnet.^25^ We aimed to guide the LLM to generate a diverse pool of lipids but also those that are relevant to constructing lipid nanoparticles. Therefore, we used two hundred molecules that were experimentally tested for EPO transfection efficiency. The prompt included the SMILES code of the lipid molecules, their molecular weight, and their transfection efficiency value. The LLM model was instructed to design 20 valid SMILES for novel, distinct, and structurally diverse molecules with high EPO transfection efficiency values and a molecular weight below 1500 kiloDaltons (kD). This query was repeated several thousand times to collect the largest possible number of unique designs. The proposed SMILES were tested for validity, converted to their canonical form, and filtered for uniqueness. Additionally, a molecular weight filter was applied to ensure the weight ranged between 500 and 1500 kDa. Molecules with cyclic structures involving more than eight atoms were also filtered out as implausible candidates.

After all the filtering steps, approximately 100,000 unique and valid molecular SMILES were obtained and merged with the current pool. To reduce the volume of the pool while maintaining its diversity, clustering was performed using the K-means algorithm. Count-based, chiral, circular fingerprints with a radius of 3 and a size of 2048 bits, calculated by the DeepChem library, were used as the numeric representations of the individual molecules. We obtained ten thousand clusters, with the most similar molecules to the centroids serving as representatives for each. See Supporting Information for more details on generation of the lipid pools.

### Gradient Boosting Classifier for Predicting Synthesizability

The CatBoost model classifies the synthesizability of lipids by leveraging an ensemble of decision trees trained on molecular fingerprints and embeddings. The model uses Extended Connectivity Fingerprints (ECFP) with a radius of 3, which essentially characterizes the molecule by counting unique substructures. The classification is performed using a gradient boosting algorithm, where the probability of synthesizability *P*(*y* = 1|*X*) given the input features *X* is calculated. The CatBoost model optimizes the following objective function:

$$L=-\frac{1}{m}\sum {i=1}^{m}\left[y_{i}\log\left(P\left(y_{i}|X_{i}\right)\right)+\left(1-y_{i}\right)\log\left(1-P\left(y_{i}|X_{i}\right)\right)\right]$$ 1

where *m* is the number of training samples, (*y_i_*) is the true label for the *i*th sample, and *P*(*y_i_* |*X*_*i*_) is the predicted probability. The model uses an ensemble of 10 classifiers with different random initializations to generate predictions for each molecule.

In each boosting iteration *m*, CatBoost:

1. Uses ordered boosting to split the data set into training subsets (folds) and to calculate the target statistics.
2. Fits a new weak learner (decision tree) to approximate the negative gradient of the loss function with respect to the current model *F*_*m*–1_(*x*).
3. Updates the model:
$$F_{m}\left(x\right)=F_{m-1}\left(x\right)+\eta \cdot f_{m}\left(x\right)$$ 2

### Active Learning

The batch variant of AL was employed, meaning that an entire batch of compounds was selected simultaneously in accordance with the setup of the labeling campaign. The primary challenge in batch selection is to identify a batch that most efficiently enhances the ML model, as selecting based on the marginal improvement of individual compounds does not guarantee optimal performance for the entire batch. For this, we extended the ALIEN method^23^ which was previously shown to be effective for chemical molecules prediction tasks.

#### Batch Selection Strategy

We extended ALIEN using a new batch selection strategy which we call *Dewdrop*. We describe here the acquisition function, i.e., the quantity we try to maximize when selecting a batch.

Given a universe U , we would like to select a candidate batch, B⊂U , with the highest joint entropy, i.e., information content. We estimate the information content in two stages:

1. First, we train the CatBoost^34^ classifier on our existing labeled data. Instead of training a single classifier to produce point predictions, we train an ensemble of 10 classifiers, with different random initializations, so that we can generate ensembles of predictions for each molecule. We use Langevin dynamics, as well as “virtual ensembles” (both described in Malinin et al.^34^) to generate ensembles of 100 predictions which approximate the “true posterior”.
2. Given ensembles of predictions for all of the candidates U , we can estimate the joint probabilities and thus joint entropy for any subset. However, binary classification of, eg., 20 molecules gives us 2^20^ ≈ 10^6^ different predictions, and 100 samples is far too few to get good estimates of all 10^6^ different joint probabilities. Therefore, we impose a higher-order independence assumption as a kind of smoothing. The *dual total correlation* of *N* random variables *X*_1_, ···, *X*_*N*_ is
$$D\left(X_{1},\cdot \cdot \cdot ,X_{N}\right)≔\overset{N}{\underset{i=1}{\sum }}H\left(\cdot \cdot \cdot ,X_{i-1},X_{i+1},\cdot \cdot \cdot \right)-\left(N-1\right)H\left(X_{1},\cdot \cdot \cdot ,X_{N}\right)$$ 3
  We next use the following simplifying assumption: **Assumption:** We assume that all dual total correlations vanish for *N* ≥ 3 (or approximately so). In this case, an argument by induction gives a simple formula for joint entropy in terms of pairwise joint entropies:
$$H\left(X_{1},\cdot \cdot \cdot ,X_{N}\right)\approx \frac{1}{N-1}\underset{i<j}{\sum }H\left(X_{i},X_{j}\right)$$ 4

Therefore, we use the ensemble of predictions to estimate pairwise joint entropies *H*(*X*_*i*_,*X*_*j*_), and then we seek batches which maximize the acquisition function

$$a\left(X_{1},\cdot \cdot \cdot ,X_{2}\right)≔\underset{i<j}{\sum }H\left(X_{i},X_{j}\right)$$ 5

In total, there were five batches of AL throughout the labeling campaign. Each batch consisted of either 20 or 40 lipid compounds, as indicated in Table 3.

### Retrospective Experiments

To evaluate the effectiveness of active learning (AL) versus random selection, we conducted retrospective experiments. In this setup, we simulated AL by withholding labels from the existing labeled data set and assessing which lipids would be selected for annotation. The training data set consisted of the initial batch and the first three batches selected by AL, while the final two AL-selected batches were reserved as the test set.

This retrospective experiment followed the structure of the original labeling campaign, with adjustments to batch size and pool sizes due to the limited number of labeled lipids. Each experiment began with an initial set of 25 lipids randomly selected from a training data set of 215 samples, with the remaining 190 samples forming the unlabeled pool. We ran 200 experiments, each with a different initial set and subsequent lipid pool. A CatBoost classifier was retrained after each successive batch selection (batch size = 10) and evaluated on a fixed test set of 42 lipids. We compared two selection strategies: the AL method proposed in this study and a random sampling baseline.

### ML Classifier Training

The CatBoost classifier was selected due to its superior performance with molecular fingerprints and LipoBart embeddings.^26^ No special hyperparameter tuning was conducted, as initial tests indicated a low dependence of test metrics on the specific values of individual hyperparameters (such as learning rate, number and depth of trees, and subsampling rate) within a sensible range. Consequently, the default parameter settings of CatBoost (version 1.2.7) were utilized throughout this study.

Featurization of molecules for AL was achieved using the Extended Connectivity Fingerprint (ECFP) with a radius of 3, employing the count-based variant that accounts for chirality. This choice was motivated by the widespread availability and acceptance of ECFP as a baseline with strong performance on small compounds and drug-like molecules, without requiring parametrization for specific areas of chemical space. Preliminary experiments validated this choice, as the performance of ECFP was comparable to that of the more advanced LipoBart embedding.

### Synthesizability Benchmarking

We evaluated the performance of our method against existing state-of-the-art synthesizability approaches using the newly developed lipid synthesizability data set, constructed through active learning (AL) and synthesizability questionnaires. Performance assessment utilized 5-fold cross-validation on the complete data set. While most comparative methods generated synthesizability scores without requiring training data, they typically produced continuous rather than binary outputs. For these continuous-output methods, we established optimal thresholds for binary classification using the training data. Specifically, we determined threshold values by maximizing accuracy on the training set, then applied these optimized thresholds to predict binary synthesizability labels for the test set.

### Refitting of DeepSA

We compared our results to those of DeepSA. The parameters for DeepSA were fitted using the code provided in their GitHub repository. The entire data set was divided into five cross-validation (CV) folds, matching exactly those used for the performance evaluation of the CatBoost classifier. Each training fold was then added to the original DeepSA training data (800,000 samples). To increase the weight of the additional data samples, they were upsampled 100 times, resulting in approximately 20,000 samples. In total, the new data constituted 2.5% of the entire training data set. All settings chosen by the authors were kept intact. The complete training of the neural network architecture was performed independently for each training fold, and performance was evaluated on the corresponding test fold. The average metrics over the five folds was reported as the final value.

### Software

AL batch selection was conducted using the Alien package (version 2.0) with the CatBoost classifier (version 1.2.7). The ECFP was calculated using the DeepChem package (version 2.8.0). The CV-fold experiments, train/test splits and performance metrics were evaluated with the scikit-learn package (version 1.5.2).

The DeepSA code was obtained from the GitHub repository at https://github.com/Shihang-Wang-58/DeepSA. The GASA code was retrieved from the repository at https://github.com/cadd-synthetic/GASA, SYBA from https://github.com/lich-uct/syba, and RAscore from https://github.com/reymond-group/RAscore. The SAScore was calculated using the RDKit package (version 2024.3.5).

## Supplementary Material



## Data Availability

Data and code for the work described in this paper is available at our github site: https://github.com/Sanofi-Public/MolQuery. Here we include a public data set curated by Moayedpour et al. which can be used to reproduce active learning retrospective experiments on LNP transfection efficiency.^26^ The synthesizability labeled data is not released due to intellectual property protection.

## Notes

Detailed description how the lipid pools generated; and performance of the refitted DeepSA using various embedding models (PDF)

## References

[1] Chaudhary N., Weissman D., Whitehead K. A. (2021). mRNA vaccines
for infectious diseases:
principles, delivery and clinical translation. Nat. Rev. Drug Discovery.

[2] Chen J., Chen J., Xu Q. (2022). Current Developments
and Challenges
of mRNA Vaccines. Annu. Rev. Biomed. Eng..

[3] Albertsen C. H., Kulkarni J. A., Witzigmann D., Lind M., Petersson K., Simonsen J. B. (2022). The role of lipid
components in lipid nanoparticles
for vaccines and gene therapy. Adv. Drug Delivery
Rev..

[4] Cui S., Wang Y., Gong Y., Lin X., Zhao Y., Zhi D., Zhou Q., Zhang S. (2018). Correlation
of the cytotoxic effects
of cationic lipids with their headgroups. Toxicology
research.

[5] Lokugamage M. P., Sago C. D., Gan Z., Krupczak B. R., Dahlman J. E. (2019). Constrained
nanoparticles deliver siRNA and sgRNA to T cells in vivo without targeting
ligands. Adv. Mater..

[6] Hou X., Zaks T., Langer R., Dong Y. (2021). Lipid nanoparticles
for mRNA delivery. Nature Reviews Materials.

[7] Chen J., Xu Y., Zhou M., Xu S., Varley A. J., Golubovic A., Lu R. X. Z., Wang K. C., Yeganeh M., Vosoughi D. (2023). Combinatorial design
of ionizable lipid nanoparticles for muscle-selective
mRNA delivery with minimized off-target effects. Proc. Natl. Acad. Sci. U. S. A..

[8] Tesei G., Hsiao Y.-W., Dabkowska A., Grönberg G., Yanez Arteta M., Ulkoski D., Bray D. J., Trulsson M., Ulander J., Lund M. (2024). Lipid shape and packing
are key for optimal design of pH-sensitive mRNA lipid nanoparticles. Proc. Natl. Acad. Sci. U. S. A..

[9] Wang S., Wang L., Li F., Bai F. (2023). DeepSA: a deep-learning
driven predictor of compound synthesis accessibility. J. Cheminf..

[10] Griffin D. J., Coley C. W., Frank S. A., Hawkins J. M., Jensen K. F. (2023). Opportunities
for Machine Learning and Artificial Intelligence to Advance Synthetic
Drug Substance Process Development. Org. Process
Res. Dev..

[11] Vaidya A., Parande D., Khadse N., Vargas-Montoya N., Agarwal V., Ortiz C., Ellis G., Kaushal N., Sarode A., Karve S. (2024). Analytical Characterization
of Heterogeneities in mRNA-Lipid Nanoparticles Using Sucrose Density
Gradient Ultracentrifugation. Anal. Chem..

[12] Ahmad, W. ; Simon, E. ; Chithrananda, S. ; Grand, G. ; Ramsundar, B. ChemBERTa-2: Towards Chemical Foundation Models, 2022. https://arxiv.org/abs/2209.01712.

[13] Ertl P., Schuffenhauer A. (2009). Estimation
of synthetic accessibility score of drug-like
molecules based on molecular complexity and fragment contributions. J. Cheminform..

[14] Kim S., Chen J., Cheng T., Gindulyte A., He J., He S., Li Q., Shoemaker B. A., Thiessen P. A., Yu B., Zaslavsky L., Zhang J., Bolton E. E. (2023). PubChem 2023 update. Nucleic Acids Res..

[15] Voršilák M., Kolář M., Čmelo I., Svozil D. (2020). SYBA: Bayesian estimation
of synthetic accessibility of organic compounds. J. Cheminf..

[16] Yu J., Wang J., Zhao H., Gao J., Kang Y., Cao D., Wang Z., Hou T. (2022). Organic compound
synthetic accessibility
prediction based on the graph attention mechanism. J. Chem. Inf. Model..

[17] Thakkar A., Chadimová V., Bjerrum E. J., Engkvist O., Reymond J.-L. (2021). Retrosynthetic
accessibility score (RAscore) – rapid machine learned synthesizability
classification from AI driven retrosynthetic planning. Chem. Sci..

[18] Genheden S., Thakkar A., Chadimová V., Reymond J.-L., Engkvist O., Bjerrum E. (2020). AiZynthFinder: a fast, robust and flexible open-source
software for retrosynthetic planning. J. Cheminf..

[19] Dorsey P. J., Lau C. L., chiun Chang T., Doerschuk P. C., D’Addio S. M. (2024). Review of machine learning for lipid
nanoparticle formulation
and process development. J. Pharm. Sci..

[20] Zendehboudi S., Rezaei N., Lohi A. (2018). Applications
of hybrid models in
chemical, petroleum, and energy systems: A systematic review. Applied Energy.

[21] Wang W., Chen K., Jiang T., Wu Y., Wu Z., Ying H., Yu H., Lu J., Lin J., Ouyang D. (2024). Artificial intelligence-driven rational design of ionizable
lipids for mRNA delivery. Nat. Commun..

[22] Venkatasubramanian V., Mann V. (2022). Artificial intelligence in reaction
prediction and chemical synthesis. Current Opinion
in Chemical Engineering.

[23] Bailey, M. ; Moayedpour, S. ; Li, R. ; Corrochano-Navarro, A. ; Kötter, A. ; Kogler-Anele, L. ; Riahi, S. ; Grebner, C. ; Hessler, G. ; Matter, H. ; Bianciotto, M. ; Mas, P. ; Bar-Joseph, Z. ; Jager, S. Deep Batch Active Learning for Drug Discovery, 2023. 10.1101/2023.07.26.550653.

[24] Wang L., Zhou Z., Yang X., Shi S., Zeng X., Cao D. (2024). The present state and challenges
of active learning in drug discovery. Drug Discovery
Today.

[25] Anthropic, A. The claude 3 model family: Opus, sonnet, haiku. Claude-3 Model Card, 2024, vol 1.

[26] Moayedpour S., Broadbent J., Riahi S., Bailey M., V Thu H., Dobchev D., Balsubramani A., ND Santos R., Kogler-Anele L., Corrochano-Navarro A. (2024). Representations of lipid
nanoparticles using large language models for transfection efficiency
prediction. Bioinformatics.

[27] Wang G., Xu S., Hu Q., Zeng F., Negishi E.-i. (2013). Search for Highly
Efficient, Stereoselective, and Practical Synthesis of Complex Organic
Compounds of Medicinal Importance as Exemplified by the Synthesis
of the C21–C37 Fragment of Amphotericin B. Chem. – Eur. J..

[28] Newhouse T., Baran P. S., Hoffmann R. W. (2009). The Economies of Synthesis. Chem. Soc. Rev..

[29] Reusch, J. Principles of Organic Synthesis – Modern Synthesis; MSU Virtual Textbook of Organic Chemistry, 2001; https://www2.chemistry.msu.edu/faculty/reusch/virttxtjml/synth2.htm.

[30] Landrum, G. RDKit: Open-source cheminformatics. https://www.rdkit.org.

[31] Aimo L., Liechti R., Hyka-Nouspikel N., Niknejad A., Gleizes A., Götz L., Kuznetsov D., David F. P., van der
Goot F. G., Riezman H., Bougueleret L., Xenarios I., Bridge A. (2015). The SwissLipids knowledgebase for
lipid biology. Bioinformatics.

[32] Xu Y., Golubovic A., Xu S., Pan A., Li B. (2023). Rational design
and combinatorial chemistry of ionizable lipids for RNA delivery. J. Mater. Chem. B.

[33] Moayedpour, S. ; Corrochano-Navarro, A. ; Sahneh, F. ; Noroozizadeh, S. ; Koetter, A. ; Vymetal, J. ; Kogler-Anele, L. ; Mas, P. ; Jangjou, Y. ; Li, S. ; Bailey, M. ; Bianciotto, M. ; Matter, H. ; Grebner, C. ; Hessler, G. ; Bar-Joseph, Z. ; Jager, S. Many-Shot In-Context Learning for Molecular Inverse Design, 2024. https://arxiv.org/abs/2407.19089.

[34] Malinin, A. ; Prokhorenkova, L. ; Ustimenko, A. Uncertainty in Gradient Boosting via Ensembles. arXiv e-prints, 2020, arXiv:2006.10562.

