# Supplementary material for: MolQuery: Prediction of Lipid Synthesizability Using Active Learning
Source: ACS Omega. 2026 Feb 11;11(7):11711–20. doi: 10.1021/acsomega.5c09931 (PMC12946983; doi:10.1021/acsomega.5c09931)
Supplement: Supplementary file 1 [file ao5c09931_si_001.pdf]

Supporting Information for MolQuery: Prediction of lipid synthesizability using active learning

Jonathan Broadbent,[†,‖] Jiří Vymětal,[‡,‖] Saeed Moayedpour,[¶,‖] Michael Bailey,[¶] Saleh Riahi,[¶] Akshay Balsubramani,[§] Peter Mikochik,[§] Luc Even,[§] Naresh Gunaganti,[§] Ramesh Dasari,[§] Hongfeng Deng,[§] Vikram Agarwal,[§] Ziv Bar-Joseph,[¶] and Sven Jager[*,¶]

†*Digital R&D, Sanofi, Toronto, ON, M5V 1V6, Canada*

‡*DataSentics, Brno, Czech Republic*

¶*Digital R&D, Sanofi, Cambridge, MA, 02141, United States*

§*mRNA Center of Excellence, Sanofi, Waltham, MA, 02451, United States*

‖*Contributed equally*

E-mail: sven.jager@sanofi.com

Creation of initial lipid pool

The initial pool of structures was created using a set of reactions that result in linkers,[?] which include esterification, Michael addition, and amide coupling. These are very commonly used in the literature to synthesize structure fragments in useful ways for LNP design. For example, esterification results in an ester linker, which behaves in relatively well-understood ways from the perspective of biodegradability and toxicity. The ester linker is by far the

most common in cationic lipid development, and a few linkers suffice to cover almost all known feasible structures.[?]

These reactions are synthesis reactions that put together fragments. In silico, we run them in reverse on a small seed set of known structures from the literature, effectively breaking each into structure fragments, at presumed linker sites only. Then, we recombine all these fragments using the same set of linker synthesis reactions used throughout, perturbing the combinations of fragments that generated the seed set. We keep combinations as possible, rejecting reaction attempts that find no appropriate structural moieties.

Even with these chemical constraints, the fragments recombine in a combinatorial number of ways that far exceeds the initial seed set of lipids. The resulting pool of structures comprises over 100,000 structures, each of which varies from a seed set molecule in one or more fragments. These constitute a rich source of structurally reasonable variation that enumerates developable chemical space as explored so far by the community.

Detailed description of LLM pool generation

The lipid pool generation involved these steps:

- Many-Shot In-Context Learning Generation[?] by LLM. The Anthropic Claude Sonnet (version "anthropic.claude-3-sonnet-20240229-v1" hosted by AWS Bedrock)[?] was used to generate SMILES based on provided example molecules with assigned experimental EPO transfection efficiency values. The prompt included instructions to ensure novelty and diversity, targeting high EPO values while limiting the molecular weight of the proposed molecules. The exact prompt used follows:

  ```
  You can generate valid SMILES for novel, different, and structurally diverse
  synthesizable lipids molecules with the desired properties.
  Examples:
      SMILES, EPO value, Molecular Weight
  ```

```
    {examples}
Task: Modify the following SMILES by combining it with fragments of the example
SMILES to generate 20 valid SMILES for novel, distinct and structurally diverse
molecules with
        1. High EPO value (above 1.2 with higher values better)
        2. Low Molecular Weight under 1500
Input SMILES: {starting_smiles}
Output format: Data in JSON format with fields "SMILES", "predicted_epo"
and "molecular_weight", no other text.
```

The placeholder {examples} is replaced by a table of 200 lipids with their associated EPO values and molecular weights. The placeholder {starting_smiles} is continuously substituted by a single SMILE string corresponding to one of approximately one hundred in-house lipid lead molecules. The query with substituted placeholders was evaluated several thousand times to gather as many distinct designs as possible.

- Filtration of Generated SMILES. The collected SMILES parsed from LLM outputs were tested for validity, converted to their canonical SMILES form, and filtered for the presence of undesired fragments. These fragments were compiled by an expert medicinal chemist during the campaign. The following list of SMARTS patterns was searched for using standard substructure search in RDKit, and any designed molecules containing these patterns were rejected. The SMARTS patterns: `CC(OC)(C)O, OC(C)OC, OC(C)(C)NC, CCCl, CCBr, CCI, N=C(O)C, [Ch1](=O), [Ch2]~[Oh1], [Nh1]~[Nh1],NNO, CSC1CN(C)CCN1C, CN1CCN(C)C(N)C1, N(O), O=C(C)OC(C)C([H])([H])O, COOC, CS(OC)(=O)=O, [H][C@@]12[C@](CCO2)([H])OCC1, CC(OC(C)=O)C([H])([H])O[H]` and `[CX3](=O)[OX2H1]`

  Additionally, a molecular weight filter was applied to ensure the values in the range between 500 and 1500 kiloDaltons. Molecules with cycles involving more than eight atoms were also filtered out.

- Merging With the Previous Version of Lipid Pool. After all the filtering steps, approximately 100,000 unique and valid molecular SMILES were obtained. This number of samples was difficult to process with the active learning algorithm, so the pool was down-sampled. To preserve diversity, clustering of the sequences in the pool was performed. We used the K-means algorithm to obtain the predetermined number of 10,000 clusters. The corresponding centroids of each cluster were then selected as representatives and preserved in the pool. The metric for distance calculation in K-means was the Euclidean distance on the circular fingerprints (2048 bits, radius 3, with chiral and counts options enabled) as calculated by the DeepChem package.

The pool generated by the above-described approach was named *llm1*. During the annotation campaign, we recognized that the *llm1* pool was overly skewed towards high-weight molecules. Therefore, the entire pool-generation process, including the clustering step, was iterated once more to increase the fraction of molecules with lower molecular weights in the pool. The LLM model was instructed to generate molecules with molecular weights below 1000 kDa, and subsequent filtration was performed within the range of 500-1200 kiloDaltons. The pool generated by this extra iteration was named *llm2*.

Table S1: Refitted DeepSA models. Performance evaluated on the lipid test set.

Encoder	Before retraining				After retraining			
	Accuracy	AUPRC	AUROC	F1	Accuracy	AUPRC	AUROC	F1
ChemMTR	0.60	0.82	0.69	0.53	0.71	0.83	0.75	0.77
ChemMLM	0.62	0.79	0.63	0.72	0.64	0.80	0.71	0.73
SmELECTRA	0.63	0.79	0.68	0.73	0.69	0.82	0.74	0.73
RoBERTa	0.55	0.74	0.57	0.60	0.70	0.83	0.76	0.76
GraphCodeBert	0.61	0.77	0.65	0.67	0.69	0.82	0.74	0.76
DeBERTa	0.57	0.78	0.62	0.51	0.76	0.86	0.79	0.80
TinBert	0.50	0.71	0.63	0.42	0.64	0.79	0.65	0.69
MinBert	0.58	0.77	0.56	0.69	0.67	0.81	0.68	0.73

Table S2: Refitted DeepSA models. Performance evaluated on the DeepSA test set.

Encoder	Before retraining				After retraining			
	Accuracy	AUPRC	AUROC	F1	Accuracy	AUPRC	AUROC	F1
ChemMTR	0.87	0.92	0.95	0.89	0.88	0.91	0.95	0.90
ChemMLM	0.87	0.91	0.94	0.89	0.87	0.91	0.95	0.89
SmELECTRA	0.86	0.91	0.93	0.88	0.87	0.91	0.94	0.88
RoBERTa	0.87	0.91	0.94	0.89	0.88	0.91	0.94	0.89
GraphCodeBert	0.88	0.92	0.94	0.89	0.88	0.91	0.94	0.89
DeBERTa	0.87	0.92	0.94	0.88	0.88	0.91	0.94	0.89
TinBert	0.86	0.91	0.94	0.88	0.87	0.91	0.94	0.88
MinBert	0.87	0.91	0.94	0.88	0.87	0.91	0.94	0.88

Table S3: Effect of ECFP parameters on performance of the CatBoost classifier. The number in parenthesis represents a standard deviation in the 5-fold CV experiment (average over 10 experiments)

radius	counted	size (bits)	chiral	accuracy	AUPRC	AUROC	F1
1	no	2048	yes	0.68(0.04)	0.83(0.06)	0.76 (0.05)	0.75 (0.05)
2	no	2048	yes	0.67(0.04)	0.82(0.07)	0.75 (0.05)	0.75 (0.04)
3	no	2048	yes	0.68(0.04)	0.83(0.06)	0.76(0.05)	0.75(0.05)
4	no	2048	yes	0.67(0.04)	0.82(0.06)	0.75(0.05)	0.74(0.04)
5	no	2048	yes	0.65(0.04)	0.81(0.07)	0.73(0.05)	0.73(0.04)
1	yes	2048	yes	0.69(0.05)	0.83(0.05)	0.76 (0.04)	0.76 (0.05)
2	yes	2048	yes	0.70(0.05)	0.84(0.06)	0.76(0.05)	0.76(0.05)
3	yes	2048	yes	0.71(0.05)	0.84(0.06)	0.77(0.05)	0.77(0.05)
4	yes	2048	yes	0.71(0.05)	0.84(0.06)	0.78(0.05)	0.78(0.04)
5	yes	2048	yes	0.71(0.05)	0.83(0.06)	0.77(0.05)	0.77(0.05)
3	yes	256	yes	0.71(0.05)	0.83(0.06)	0.77(0.05)	0.77(0.05)
3	yes	512	yes	0.71(0.05)	0.84(0.06)	0.78(0.05)	0.78(0.05)
3	yes	1024	yes	0.71(0.04)	0.84(0.06)	0.77(0.05)	0.78(0.04)
3	yes	4086	yes	0.71(0.05)	0.83(0.06)	0.77(0.05)	0.78(0.05)
3	yes	8192	yes	0.71(0.05)	0.83(0.06)	0.77(0.05)	0.77(0.05)
1	yes	2048	no	0.69(0.04)	0.83(0.05)	0.76(0.04)	0.76(0.04)
2	yes	2048	no	0.68(0.05)	0.83(0.06)	0.75(0.05)	0.75(0.04)
3	yes	2048	no	0.69(0.04)	0.83(0.06)	0.76(0.05)	0.76(0.04)
4	yes	2048	no	0.69(0.05)	0.83(0.06)	0.77(0.05)	0.76(0.05)
5	yes	2048	no	0.69(0.04)	0.84(0.06)	0.77(0.05)	0.76(0.04)

Table S4: Effect of chosen parameters of the CatBoost classifier on its performance. The number in parenthesis represents a standard deviation in the 5-fold CV experiment (average over 10 experiments)

parameter	value	accuracy	AUPRC	AUROC	F1
iterations	500	0.71(0.05)	0.84(0.06)	0.77(0.05)	0.77(0.05)
iterations	1000(default)	0.71(0.05)	0.84(0.06)	0.77(0.05)	0.77(0.05)
iterations	2000	0.71(0.05)	0.84(0.06)	0.77(0.05)	0.77(0.05)
learning rate	0.0025	0.72(0.05)	0.83(0.06)	0.77(0.05)	0.78(0.05)
learning rate	0.005237 (default - automatic)	0.71(0.05)	0.84(0.06)	0.77(0.05)	0.77(0.05)
learning rare	0.010	0.71(0.05)	0.83(0.06)	0.77(0.05)	0.77(0.05)
depth	4	0.72(0.05)	0.83(0.06)	0.77(0.05)	0.78(0.05)
depth	5	0.71(0.05)	0.84(0.06)	0.77(0.05)	0.78(0.05)
depth	6 (default)	0.71(0.05)	0.84(0.06)	0.77(0.05)	0.77(0.05)
depth	7	0.71(0.05)	0.84(0.06)	0.77(0.05)	0.77(0.05)
depth	8	0.71(0.05)	0.84(0.06)	0.77(0.05)	0.77(0.05)

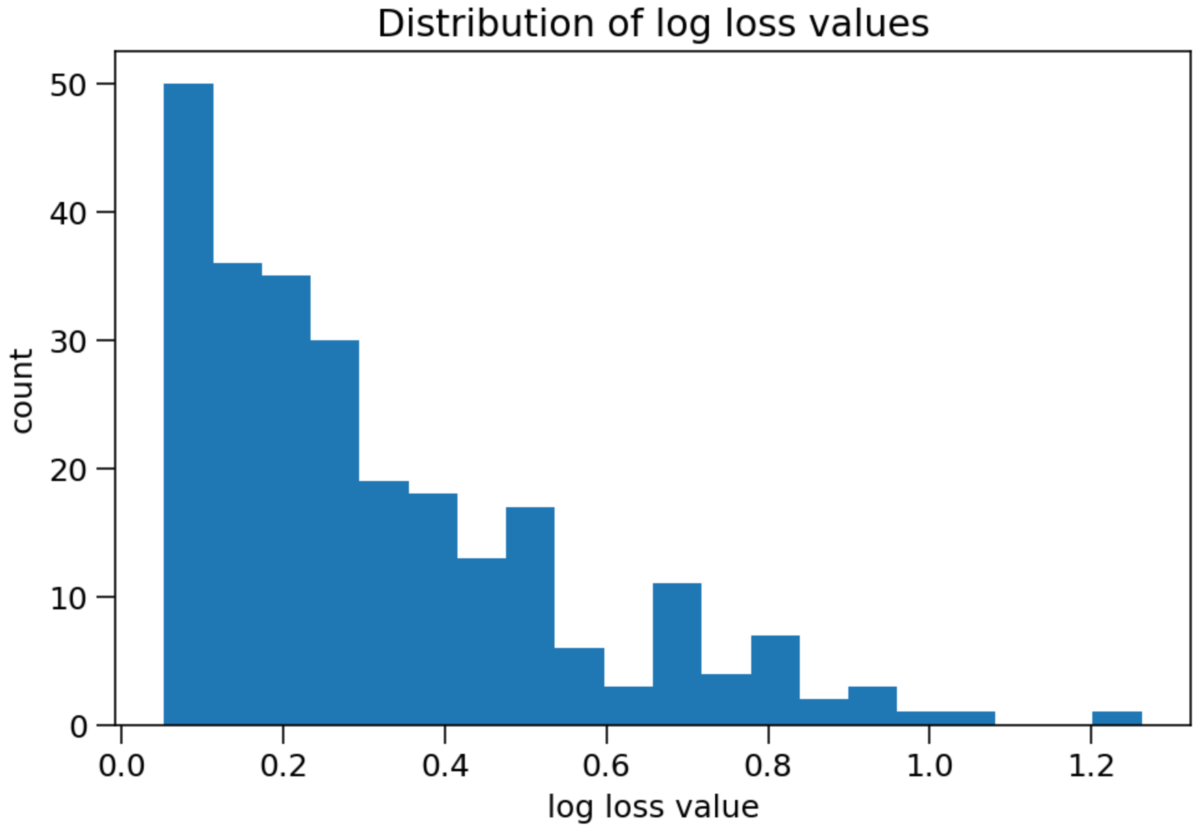

Figure S1: Distribution of log loss function values for individual training data samples. The log loss values were obtained after training the CatBoost classifier on the entire dataset. Higher values indicate greater deviation from the ground truth.

Additional Discussion

Capturing Long-Range Molecular Information

In the case of ECFPs, this ability is limited by the radius property of the underlying fragmentation algorithm. For the radius of 3 used in this study, it follows that the link between atoms separated by more than six bonds cannot be directly captured. We found several cases of fingerprint conflict in our lipid dataset, where the same fingerprint was generated from different lipid molecules. Consequently, the ML model is unable to distinguish between these molecules. Graph neural networks can suffer from a similar problem due to the ef-

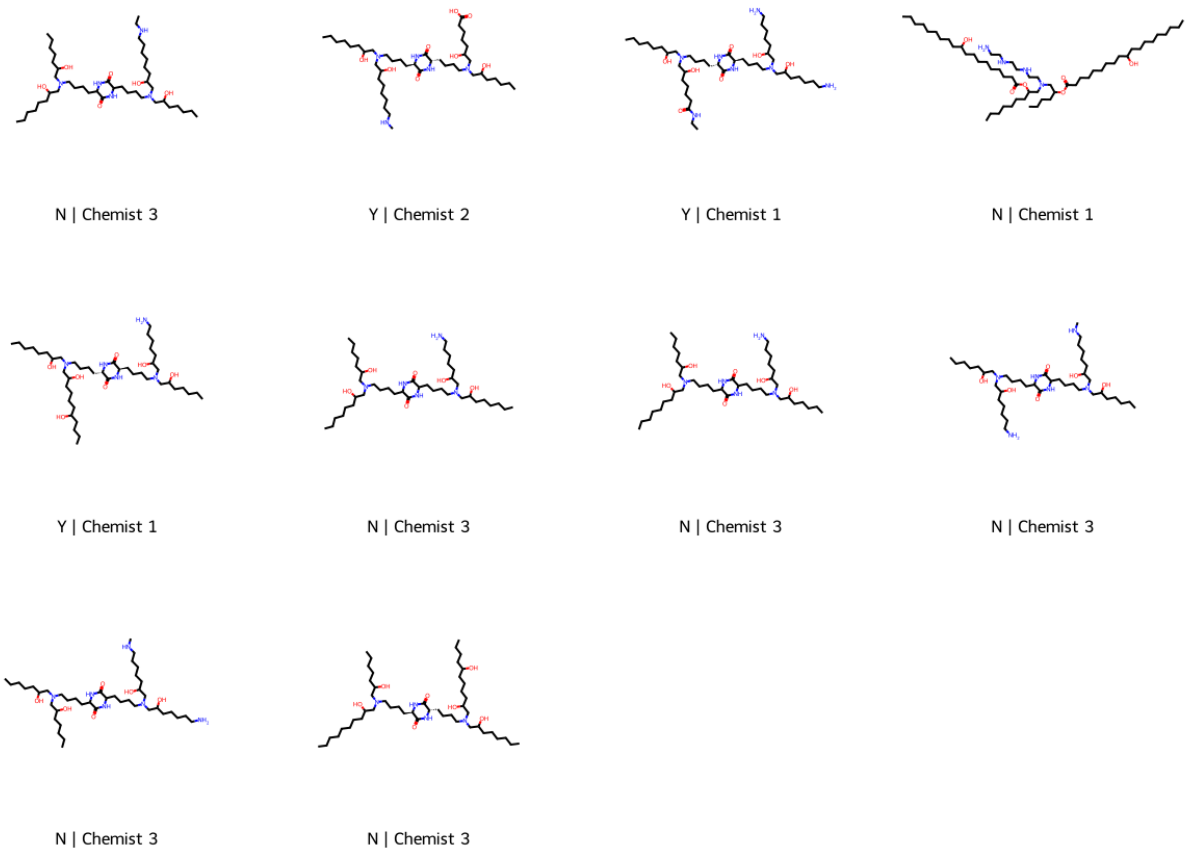

Figure S2: Synthesizability outliers. Top ten molecules with the highest log loss values. Annotations show the label and which chemist provided the annotation.

fect known as “over-squashing,” where nodes become insensitive to information from distant nodes. Modern language models, on the other hand, should not suffer from this effect due to the attention mechanism. Nevertheless, it is difficult to analyze whether such information is leveraged for prediction.

Intrinsic difficulty of the synthetic accessibility prediction

Predicting synthetic accessibility is notoriously difficult and so the performance we observed may actually be close to the limit of current AI technologies given available training data. Molecules can contain numerous functional groups, each with distinct reactivity patterns. The presence and arrangement of these groups can significantly influence the synthetic route and may necessitate additional steps, such as the introduction of protective groups. Synthetic accessibility is further influenced by factors such as the availability of starting materials, reaction conditions, and the expertise of the chemist. Even chemically similar molecule,s located close in chemical space, might require completely different synthetic pathways.
